# Nanotechnology in cancer treatment: revolutionizing strategies against drug resistance

**DOI:** 10.3389/fbioe.2025.1548588

**Published:** 2025-04-30

**Authors:** Shazia Sabir, Ali Salman Bin Thani, Qamar Abbas

**Affiliations:** Department of Biology, College of Science, Sakhir Campus, University of Bahrain, Sakhir, Bahrain

**Keywords:** nanoparticles, nanomaterials, drug delivery, targeted strategies, nanotechnology, cancer, diagnosis, treatment

## Abstract

A notable increase in cancer-related fatalities and morbidity worldwide is attributed to drug resistance. The factors contributing to drug resistance include drug efflux via ABC transporters, apoptosis evasion, epigenetic alterations, DNA repair mechanisms, and the tumor microenvironment, among others. Systemic toxicities and resistance associated with conventional cancer diagnostics and therapies have led to the development of alternative approaches, such as nanotechnology, to enhance diagnostic precision and improve therapeutic outcomes. Nanomaterial, including carbon nanotubes, dendrimers, polymeric micelles, and liposomes, have shown significant benefits in cancer diagnosis and treatment due to their unique physicochemical properties, such as biocompatibility, stability, enhanced permeability, retention characteristics, and targeted delivery. Building on these advantages, this review is conducted through comprehensive analysis of recent literature to explore the principal mechanisms of drug resistance, the potential of nanomaterials to revolutionize selective drug delivery and cancer treatment. Additionally, the strategies employed by nanomaterials to overcome drug resistance in tumors, such as efflux pump inhibition, multidrug loading, targeted delivery to the tumor microenvironment, and gene silencing therapies are discussed in detail. Furthermore, we examine the challenges associated with nanomaterials that limit their application and impede their transition to clinical use.

## 1 Introduction

Cancer therapies are often compromised due to the significant challenge of drug resistance. This resistance arises from multiple factors, including drug deactivation, changes in drug targets, evasion of apoptosis, drug efflux, DNA damage repair, cell heterogeneity, and immune system escape within the tumor microenvironment (TME) (Li Y. et al., 2021). Several transporters, expressed in various tissues, contribute to drug resistance by translocating numerous substances, including drugs, across the plasma membrane (Catalano et al., 2022). Genetic instability is another major factor that leads to reduced cell differentiation and maturation, with poor contact inhibition resulting in the transition from benign to aggressive malignancies (Khalaf et al., 2021). Moreover, alterations in the lipid composition and synthesis of the plasma membrane play a critical role in the development of drug resistance phenotypes. These changes hinder intracellular drug transportation and reduce the cytotoxic effects of chemotherapy (Haider et al., 2020). The complexity of these resistance mechanisms underscores the necessity for targeted therapies to manage drug resistance in cancer treatment (Nussinov et al., 2021) (Table 1). Therefore, a molecular understanding of drug resistance is vital for the successful treatment of cancers (Luque-Bolivar et al., 2020).

**TABLE 1:** Key barriers to drug resistance in cancer therapy.

Key barrier	Description
Drug deactivation	Breakdown or modification of the drug, reducing its effectiveness
Changes in drug targets	Alterations in cellular targets that drugs aim to attack
Apoptosis obstruction	Prevention of programmed cell death, allowing cancer cells to survive
Drug efflux	Transporters expel drugs out of cells, lowering their intracellular concentration
DNA damage repair	Enhanced repair mechanisms negate the effects of DNA-damaging drugs
Cell heterogeneity	Genetic and phenotypic variability among cancer cells hinders uniform treatment efficacy
Immune system evasion	TME enables cancer cells to avoid detection and destruction
Genetic instability	Results in poor cell differentiation and aggressive malignancies
Lipid layer modification	Changes in lipid composition of plasma membrane hinder intracellular drug transport

Nanotechnology, which involves the use of drug-loaded nanomaterials (NMs), has emerged as a highly advanced technique to significantly enhance the aggregation of cancer drugs at the targeted site (Salem et al., 2023). It plays a crucial role in minimizing widespread toxicity and overcoming drug resistance, thanks to its enhanced biodistribution, tissue compatibility, and stability (Liu et al., 2017). Despite their small size (1–100 nm), NMs provide a large surface-to-volume ratio for biomolecule assembly, thus increasing the potency of the drug (Cao et al., 2021). A major milestone in the revolution of pharmacology was marked in 1995 with the FDA approval of polyethylene glycolated liposomal doxorubicin (Doxil), which is now used for treating several cancers (Barenholz, 2012). NMs are designed according to the tumor’s etiology to effectively combat drug resistance. Significant progress has been made in overcoming drug resistance in cancers such as breast, ovarian, and prostate cancer (Yao et al., 2020). Liposomes, polymeric micelles, dendrimers, and inorganic nanoparticles are among the developed NMs used in this context (Emran et al., 2022). These NMs create intelligent systems that react to various external and internal stimuli, such as temperature, pH, enzymes, optics, and magnetism. Depending on the nature of the treatment, suitable NMs are selected based on criteria like size, shape, and physicochemical properties (Table 2). For example, micelles can deliver hydrophobic and bipolar drugs, while gold nanoparticles and liposomes are ideal for cellular uptake in the liver and blood (Kim and Khang, 2020). Moreover, the use of artificial intelligence to design NMs is a cutting-edge development that further enhances the sophistication and potential of these innovative nanoscale technologies (Sun et al., 2023). Nanomedicine boosts drug efficacy by selectively targeting and destroying cancer cells. However, NM-based therapies present challenges, such as the need for extensive preclinical assessments, rigorous clinical trials, and specific clinical validations to ensure the successful translation of these drugs (Wahab et al., 2021). Furthermore, understanding the molecular mechanisms of NMs in overcoming drug resistance is crucial for advancing the development of biocompatible materials and tailored nanomedicines. The key advantages of nanoparticulate formulations over conventional solution forms such as enhanced solubility, improved targeting, and prolonged circulation are summarized in Table 3 (Ray et al., 2021).

**TABLE 2:** Comparison of synthetic procedures for various nanomaterials used in cancer therapy.

Nanomaterial type	Description	Method	Advantages	Disadvantages	Applications and findings	Citations
Liposomes	Liposomes are lipid-based nanocarriers used for targeted drug delivery, leveraging unique properties like small size, high surface area, and biocompatibility	Liposomes are designed to encapsulate hydrophilic and hydrophobic drugs, targeting specific cells via surface modifications (e.g., ligands)	Small size, high surface area, biocompatibility, targeted delivery, and controlled release	Long-term stability concerns, potential immunogenicity, and issues with large-scale production	Liposomes like Rg3-PTX-LPs target resistant breast cancer cells and reverse drug resistance, achieving significant tumor inhibition. ApoA1-modified cationic liposomes enhance doxorubicin uptake and minimize adverse effects	Majumder and Minko, 2021; An et al., 2021
SLNs (solid lipid nanocarriers)	SLNs encapsulate both hydrophilic and hydrophobic drugs, enhancing drug solubility, bioavailability, and controlled release	SLNs are formulated from solid lipids and surfactants, which can be modified to improve drug loading and stability	Enhanced solubility and bioavailability, controlled release, and biocompatibility	Challenges with moisture content, poor drug loading, and stability issues during storage	SLNs like C-peptide-SLNs enhance paclitaxel cytotoxicity against resistant cancer cells. Dual-aptamer-functionalized SLNs improve drug delivery and therapeutic efficacy. SLNs face challenges like high moisture content and poor stability, addressed by NLCs	Rahdari et al., 2025; Darabi et al., 2022
NLCs (nanostructured lipid carriers)	NLCs are a second-generation lipid nanoparticle system designed to address stability and drug-loading issues of SLNs	NLCs are created by mixing solid lipids and liquid lipids, improving stability and drug loading capacity	Improved stability, enhanced drug-loading capacity, prolonged circulation, and reduced degradation	Potential for rapid clearance, off-target effects, and challenges with scalability	NLCs improve stability and drug loading, offering prolonged circulation, targeted delivery, and reduced degradation. Applications like Nano Repair Q10 cream demonstrate their potential in drug delivery	Sheoran et al. (2022)
Polymeric micelles	Self-assembled, biphasic structures with a hydrophilic outer shell and a hydrophobic core, ideal for drug delivery, improving solubility, retention, and minimizing side effects	Polymeric micelles are formed by amphiphilic block copolymers in aqueous solutions, forming stable structures that encapsulate both hydrophilic and hydrophobic drugs	Improves drug solubility, retention, targeted delivery, and minimizes systemic side effects	Long-term effects on glucose metabolism in normal tissues, challenges in scalability, and regulatory approval for clinical use	Polymeric micelles improve drug solubility and retention, enhancing therapeutic efficacy while targeting tumor accumulation. Effective in delivering resveratrol and reducing systemic toxicity in breast cancer cells. Challenges include clinical translation and long-term effects	Lee et al., 2022; Gregoriou et al., 2020
Dendrimers	Branched macromolecules with a core-shell structure, highly versatile for drug delivery and drug resistance management	Dendrimers are synthesized using controlled polymerization techniques, leading to highly branched structures capable of encapsulating drugs and targeting specific tumor sites	Highly branched structure for efficient drug encapsulation, versatile for drug delivery, and capable of overcoming MDR by downregulating MDR genes	Surface charge-dependent toxicity, potential issues with biocompatibility and long-term safety in clinical applications	Dendrimers like PAMAM enable targeted drug delivery, co-delivery of siRNA, and overcoming MDR by downregulating P-glycoprotein. Their biocompatibility and ability to restore drug sensitivity are key for treating resistant cancers. Challenges include toxicity and surface charge	Gavas et al., 2021; Zhang et al., 2021
Carbon nanotubes (CNTs)	CNTs possess unique mechanical, electrical, and optical properties, allowing them to effectively deliver drugs, penetrate cell membranes, and target tumor cells	CNTs, particularly multi-walled CNTs (MWCNTs), can be loaded with drugs and functionalized for tumor targeting. Magnetically controlled CNTs (mCNTs) offer precise, non-invasive drug delivery	Efficient drug delivery, ability to target tumor cells and intracellular sites, non-toxic magnetic manipulation for therapy	Production scalability, potential immune responses, and long-term stability in clinical applications remain challenging	CNTs effectively reverse drug resistance by targeting drug-resistant pathways, such as EMT, in cancers like glioblastoma and non-small cell lung cancer (NSCLC). Their ability to be magnetically controlled improves treatment efficacy	Tang et al., 2021; Wang et al., 2023; Qi et al., 2021; Kamazani et al., 2021
Fullerenes	Fullerenes (C60) have antioxidative properties, enhance ROS production, and are utilized in photodynamic therapy for cancer	Fullerenes are used as photosensitizers or as part of drug nanocomposites to enhance the delivery and cytotoxicity of anticancer drugs	Ability to generate ROS, enhance drug delivery, and selectively target resistant cancer cells	Toxicity concerns, challenges in functionalization, and stability during storage	Fullerenes like C60 nanoparticles enhance therapeutic efficacy by increasing ROS production and targeting resistant cancer cells. Functionalized fullerenes show promise in photodynamic therapy and combinational treatments	Fawal et al., 2023; Horak et al., 2021; Fu et al., 2024
Graphene	Graphene and graphene oxide (GO) possess high biocompatibility and are highly effective in delivering drugs and generating ROS to overcome drug resistance in cancer therapy	Graphene oxide is often functionalized and used to deliver drugs such as doxorubicin, combined with pH-sensitive release mechanisms	Excellent drug delivery, biocompatibility, ability to generate ROS for therapeutic effects, and ability to target tumor sites	Cytotoxicity at higher doses, difficulty in controlling functionalization, and scalability for clinical applications	Graphene oxide is used for targeted drug delivery in multidrug-resistant cancers, including glioma. Its ability to generate ROS and target specific cancer cells holds great promise in overcoming resistance	Zhang et al., 2022
Iron nanoparticles	Iron nanoparticles, especially porous magnetic hematite (Fe_2_O_3_), induce ferroptosis, an iron-dependent cell death mode, particularly effective for drug-resistant cancer cells	Iron nanoparticles can load anticancer drugs like doxorubicin without coatings, and their magnetic properties allow for precise, targeted delivery	Induces ferroptosis, effective against drug-resistant cancers, magnetic targeting, and biocompatibility	Long-term stability, bio-corona formation, and regulatory challenges in clinical applications	Iron nanoparticles like Fe_2_O_3_-loaded doxorubicin carriers induce apoptosis in resistant cancer cells and demonstrate superior cytotoxicity. Synergistic approaches combining ferroptosis induction with PDT and MWDT enhance efficacy	Sheikh et al., 2024; Ansari et al., 2022; Zhou et al., 2022
ZnO nanoparticles	ZnO-NPs exhibit antitumor properties, inducing apoptosis and enhancing chemotherapy drug effects, though their role in overcoming resistance in some cancers remains unclear	ZnO-NPs can be modified to respond to specific tumor conditions, enabling enhanced drug delivery and targeting in MDR cancers	Induces apoptosis, enhances chemotherapy drug effects, and improves targeting in MDR cancers	Concerns about drug resistance in certain cancers and surface functionalization issues	ZnO-NPs enhance anticancer effects in MDR tumors, e.g., breast cancer, by improving doxorubicin delivery. Some variants show resistance mechanisms	Zhou et al., 2023; Thakral et al., 2023; Wu et al., 2022
Silver nanoparticles	Silver nanoparticles (AgNPs) exhibit apoptosis-inducing properties, with biocompatible versions showing minimal toxicity to normal cells while retaining efficacy in tumor cells	AgNPs induce apoptosis through ROS generation, with efficacy varying with concentration and time	Strong anticancer potential, induces apoptosis, and minimal toxicity towards normal cells	Concerns about the cytotoxicity and concentration-dependent effects in long-term use	AgNPs induce DNA condensation and apoptosis in drug-resistant tumor cells, particularly MDA MB-231. Biologically synthesized AgNPs have minimal toxicity toward normal erythrocytes	Oves et al., 2022; Gomes et al., 2021; Swilam and Nematallah 2020
Gold nanoparticles	Gold nanoparticles (AuNPs) enhance targeted drug delivery and overcome resistance in cancers, offering low toxicity and modulated drug release through functionalization	AuNPs are synthesized and functionalized with ligands for precise drug delivery and targeted release	High biocompatibility, non-immunogenic, enhanced drug delivery, and ability to overcome resistance	Concerns about stability, long-term toxicity, and scalability in clinical settings	AuNPs overcome glioblastoma resistance to TMZ through enhanced cellular uptake and apoptosis. Their biocompatibility and safety in drug delivery hold promise for cancer therapy	Soliman et al., 2023; Perveen et al., 2021; Y. Yu et al., 2022
Quantum dots	Quantum dots (QDs) are semiconducting nanomaterials with optical properties, effective in overcoming drug resistance through ROS production and targeted drug delivery	QDs are conjugated with drugs, antibodies, and adjuvants, enhancing drug delivery and addressing MDR through gene regulation	ROS generation, targeted drug delivery, and effective in MDR cancers	Concentration-dependent toxicity and challenges in precision targeting in clinical settings	Quantum dots, particularly graphene-based, overcome MDR by regulating drug resistance genes and enhancing drug retention. Their potential in targeting cancer cells is constrained by toxicity	Yadav et al., 2022

**TABLE 3:** Key benefits of nanoparticulate formulations compared to solution forms.

Property	Solution form	Nanoparticulate form (non-targeted)	Nanoparticulate form (targeted)
Bioavailability	Low	Moderate to high	High
Drug stability	Rapid degradation	Improved stability	Enhanced stability and retention
Targeted delivery	Non-specific distribution	Passive accumulation (EPR effect)	Active targeting via ligands
Side effects	High systemic toxicity	Reduced systemic toxicity	Minimal off-target effects
Circulation time	Short	Extended (PEGylation)	Extended (ligand-mediated targeting)
Therapeutic efficiency	Low	Moderate	High

**FIGURE 1:**
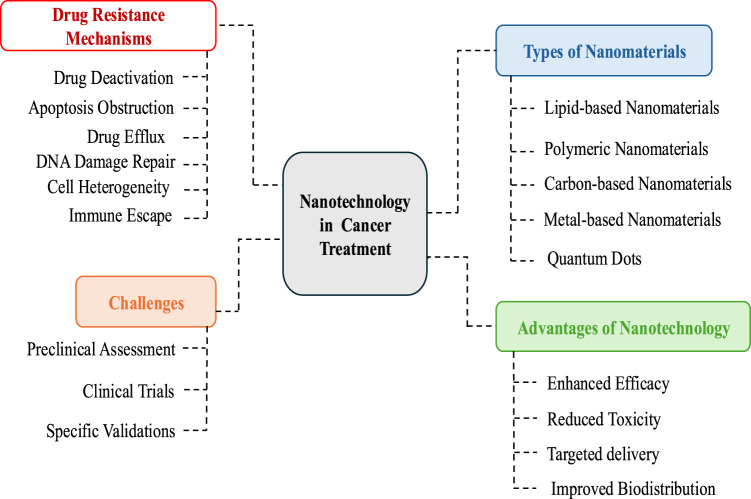
Schematic presentation of role of nanmaterials against drug resistance in cancer treatment.

Despite the wealth of publications on nanotechnology in cancer therapy, most research focuses primarily on nanoparticle synthesis and application. Therefore, a comprehensive review of the latest literature is essential to understand the current state of research and identify recent advancements. The objectives of this article are to analyze the mechanisms of drug resistance in cancer therapy, summarize the role of various nanomaterials in overcoming drug resistance, and evaluate recent advancements in nanoparticle-based drug delivery systems to combat resistance. The review also aims to identify the challenges faced in this field and propose future research directions to enhance the clinical translation of nanomedicine. Unlike previous studies that focus on specific nanoparticles, this review provides a holistic synthesis of various nanomaterials (lipid-based, polymer-based, carbon-based, and metal-based) and their applications. Additionally, it highlights gaps in understanding nanoparticle interactions within the tumor microenvironment and their long-term biological effects, offering valuable insights for future research. The study also addresses emerging advancements in stimuli-responsive nanoparticles and the co-delivery of nanomaterials, which contribute to the optimization of nanomedicine for clinical applications (Figure 1).

## 2 Mechanisms of tumor drug resistance

Tumor drug resistance presents a significant challenge for therapeutic agents through numerous mechanisms that allow cancer cells to evade apoptosis, autophagy, and regular signaling pathways. One key mechanism of drug resistance is the reduction of drug concentration and efficacy through higher efflux pump activity, such as P-glycoprotein (P-gp). Additionally, tumors acquire drug resistance through mutations in the genes encoding these targets, rendering the drugs less effective or completely ineffective. Epigenetic modifications, such as irregular DNA methylation and histone changes, also contribute to drug resistance by altering gene expression and fostering cell survival.

**FIGURE 2:**
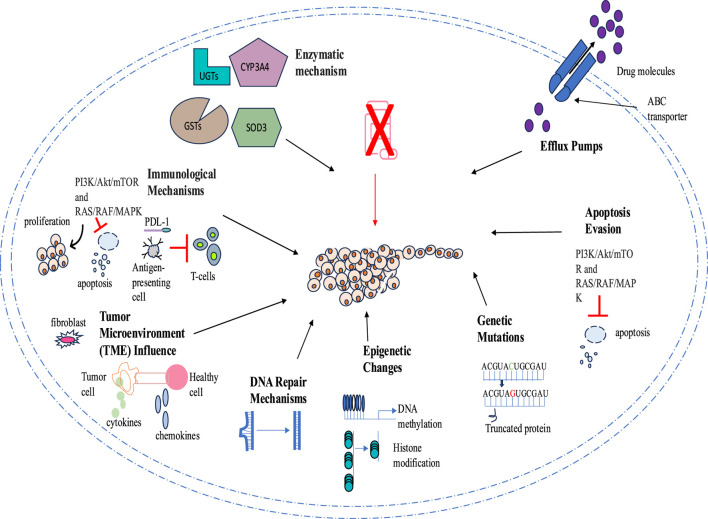
Mechanism of drug resistance in cancer cells.

Another mechanism involves cellular repair pathways that counteract drug-induced damage; for example, enhanced DNA repair processes can correct the genetic modifications induced by chemotherapy. The tumor microenvironment (TME) makes cancerous cells less vulnerable to drugs by creating a protective niche. Therefore, the intricate nature of resistance mechanisms necessitates combination therapies that simultaneously target multiple pathways to improve therapeutic effectiveness. A comprehensive understanding of these mechanisms is essential to combat drug resistance in cancer therapies (Figure 2).

### 2.1 ABC transporters in cancer drug resistance

Based on genome sequences and transmembrane domains, seven subfamilies (A to G) of ABC transporters have been identified, comprising 49 proteins. Of these, 19 are believed to participate in the efflux of anticancer drugs (Juan-Carlos et al., 2021; Wang et al., 2021). Drug efflux ABC transporters, such as P-gp, multidrug resistance protein-1 (MRP-1), and breast cancer resistant protein (BCRP, MXR, ABCG2), play a crucial role in various cancers due to their distinct structural and functional characteristics. ABC transporters like P-glycoprotein (P-gp), MRP1, and BCRP mediate multidrug resistance by actively expelling chemotherapeutic drugs from cancer cells using ATP hydrolysis-driven conformational changes. This process reduces intracellular drug concentrations, making cancer cells less responsive to treatment. Moreover, the expression of multiple ABC transporters can occur within a single tumor. For example, in 281 cases of acute myeloid leukemia, 49 different transporter proteins were found to be associated. Additionally, there is strong evidence of a correlation between the coexpression of transporter genes and patient survival (Pote and Gacche, 2023). Elevated drug doses to induce apoptosis become ineffective due to the crucial role of ABC transporter proteins in helping cells evade programmed cell death. These transporter proteins significantly affect drug bioavailability and intracellular levels, as well as traverse the blood-brain barrier, thereby limiting drug delivery. While increasing drug concentration is a common strategy to overcome this challenge, it often leads to systemic toxicity. Consequently, enhanced drug efflux is a principal mechanism of drug resistance in cancer cells against treatments (Bukowski et al., 2020). Several ABC transporters are significant in less differentiated cancer variants and metastatic sites. Inhibiting these transporters has emerged as a potential strategy to address this issue. Novel analeptics are being developed to enhance drug sensitivity, initiate programmed cell death, and minimize cell survival, considering the effects of these molecules. ABCB1 and ABCG2 are important targets for improving therapeutic efficacy. Their expression is regulated by genetic polymorphisms and non-coding RNAs, making them key targets for overcoming MDR. Strategies to counter MDR include ABC transporter inhibitors, alternative therapeutics that bypass efflux mechanisms, and gene silencing techniques to restore drug sensitivity in resistant cancer cells (Modi et al., 2022). A study by C. P. Wu et al. (2020) showed that Sitravatinib suppresses the activity of ABCB1 and ABCG2, preventing drug efflux and allowing higher intracellular drug accumulation. By restoring cancer cell sensitivity to chemotherapy, it enhances drug retention and improves treatment efficacy in resistant tumors. However, no inhibitors for ABCB1 and ABCG2 have yet been approved by the FDA for this purpose (C. P. Wu et al., 2020). Therefore, future research should prioritize overcoming the limitations of existing ABC transporter inhibitors and developing innovative, more effective strategies to counteract ABC transporter-mediated drug resistance in cancer. Utilizing stimuli-responsive nanoparticles and co-delivering transporter inhibitors may improve drug retention and therapeutic efficiency while minimizing systemic toxicity.

### 2.2 Apoptosis evasion in cancer drug resistance

The fundamental characteristic of malignant tumors is the regulation of apoptotic pathways, which gives neoplastic cells a propensity to resist programmed cell death. The B-cell lymphoma-2 (Bcl-2) family, members of the inhibitory apoptotic proteins family, as well as their regulator, the tumor suppressor p53, and the overactivation of the PI3K/AKT pathway, are crucial in contributing to drug resistance in several tumors (Neophytou et al., 2021). The balance between cell viability and apoptosis at various stages of each pathway is regulated by diverse regulatory molecules, including FLIP, Bcl-2 superfamily proteins, and inhibitory apoptotic proteins in each cell. Death receptors, such as Fas ligand and tumor necrosis factors, activate caspase-8 through the extrinsic apoptotic pathway, while the intrinsic mitochondrial pathway releases cytochrome c to activate caspase-9. Ultimately, apoptosis is executed by terminal caspases, such as caspase-3, following the convergence of both extrinsic and intrinsic pathways at terminal caspases. However, in tumor cells, apoptosis is thwarted by the inhibitor of apoptosis protein Survivin at caspase-9 instead of caspase-3 (Grossman and Altieri, 2001). Moreover, metabolic reprogramming also plays a significant role in the development of drug resistance in cancer cells. For instance, resistant cancer cells preferentially utilize glycolysis instead of oxidative phosphorylation for ATP production through the Warburg effect, which promotes survival and evasion of drug-induced apoptosis (Shi et al., 2024). Additionally, resistant cancer cells utilize alternative metabolic substrates such as glutamine and lipids to sustain survival during treatment (Cioce et al., 2024). Cancer stem cells further enhance resistance by exhibiting metabolic plasticity, shifting between glycolysis and oxidative phosphorylation in response to environmental conditions (Masoudi et al., 2024). Due to the complex regulation of apoptosis and its frequent disruption in cancer, a comprehensive understanding of the molecular and metabolic mechanisms that control apoptosis is essential for developing effective therapeutic strategies.

### 2.3 Genetic mutation in cancer drug resistance

Gene mutation, gene amplification, and non-coding mutations are significant contributors to cancer cell resistance. Resistance due to specific point mutations enhances cell survival under treatment and increases drug dependency (Coelho et al., 2024). For instance, mutations in the epidermal growth factor receptor (EGFR), such as the p.T790M mutation, and the MPS1 gene confer resistance to EGFR inhibitors like gefitinib and MPS1 inhibitors, including AZ3146, NMS-P715, and CCT251455, respectively (Gurden et al., 2023). This type of resistance is commonly associated with rapid tumor recurrence due to the swift propagation of genetic alterations within the tumor population. While gene amplification resistance leads to slower tumor recurrence, it also results in more aggressive and diverse tumor populations through increased gene copy number, which drives the evolution of more resistant clones. The selection of these mechanisms is determined by the frequency of mutation and amplification events, along with the number of alterations required to establish resistance (Li and Foo, 2023; Lin et al., 2023). A recent study by Jia et al. (2024) revealed adaptive resistance due to secondary mutations that develop after treatment. For example, mutations in homologous recombination repair genes like BRCA1 and BRCA2 contribute to resistance against platinum-based chemotherapy and poly (ADP-ribose) polymerase inhibitors (Jia et al., 2024). Moreover, non-coding mutations, such as those at Top1 cleavage sites, confer resistance to the topoisomerase I inhibitor irinotecan by reducing drug-induced DNA damage and lowering the cytotoxic effects of the drug (Kumar et al., 2023). These findings emphasize the need to consider non-coding regions alongside adaptive resistance and naturally occurring resistance, given the inherent mutagenic plasticity of cancer. This underscores the necessity for personalized cancer management strategies.

### 2.4 Epigenetic alteration in cancer drug resistance

Epigenetic processes of DNA methylation and histone modification, as well as microRNAs (miRNAs), play a vital role in various types of cancer. Epigenetic drug resistance in cancer cells often arises from mutations in key components of the Polycomb repressive complex 2 (PRC2), which lead to the reestablishment of condensed chromatin structures and increased levels of H3K27me3, thereby silencing tumor suppressor genes (TSGs). Additionally, mutations in genes such as TET2 or elevated expression of DNMT3A can contribute to *de novo* DNA methylation, further promoting chromatin recondensation and resistance to therapies like valemetostat. These adaptive changes in the epigenome allow cancer cells to escape the effects of targeted treatments, highlighting the need for strategies that address these epigenetic alterations (Yamagishi et al., 2024). Histone deacetylation plays a significant role in cancer cell metabolism. This process inhibits gluconeogenesis and promotes glycolysis, a phenomenon known as the Warburg effect. Increased glycolysis and lactic acid production are linked to resistance against tyrosine kinase inhibitors (TKIs) like Osi. Specifically, histone deacetylase (HDAC) is involved in the differentiation of M2 macrophages, which are associated with tumor progression and drug resistance (Lin et al., 2023). DNA methylation is crucial for the maturation of cancer cells, including programmed cell death, chromatin remodeling, DNA repair, metabolism, regulation of transcription, and the cell cycle. DNA methylation plays a crucial role in mediating EMT and resistance to cancer treatments. Galle et al. (2020) suggest that monitoring DNA methylation changes in circulating tumor DNA (ctDNA) could serve as a non-invasive method to predict resistance to treatments like sorafenib in patients, highlighting the clinical implications of these mechanisms. Specifically, changes in DNA methylation patterns can lead to the silencing of epithelial markers and the activation of mesenchymal markers, which are associated with a more aggressive cancer phenotype. Furthermore, methylation-driven EMT was observed in cell lines resistant to various chemotherapies and targeted agents, including 5-fluorouracil and sorafenib. This suggests that the underlying mechanisms of resistance may be common across different types of cancer treatments (Galle et al., 2020). Liu et al. (2022) investigated the role of DNA methyltransferase 1 (DNMT1) in breast cancer by examining its influence on the miR-497/GPRC5A axis, noting abnormal DNA methylation in tumor suppressor gene promoters. The experimental study found that increased DNMT1 expression promotes chemotherapy resistance and metastasis by repressing miR-497 and enhancing GPRC5A expression through methylation of its promoter. However, *in vivo* studies showed that reducing DNMT1 levels can inhibit breast cancer growth and spread, highlighting its potential as a therapeutic target (Liu et al., 2022). Considering the critical roles of epigenetics in drug resistance, continued research in this field has the potential to uncover new therapeutic strategies and improve patient outcomes.

### 2.5 DNA repair mechanisms in cancer drug resistance

DNA repair mechanisms play a crucial role in maintaining genome stability by correcting damaged DNA and reducing the risk of carcinogenesis. For instance, mutations in DNA repair-related genes, such as BRCA1 and BRCA2, are linked with elevated levels of breast, ovarian, and pancreatic cancers. Moreover, DNA-dependent protein kinase, a key enzyme involved in repairing double-strand breaks via the non-homologous end-joining pathway, can induce cell death and genetic mutations (Li L. Y. et al., 2021). Similarly, mutations or epigenetic alterations in human mutS homologs 2 (hMSH2) and 6 (hMSH6) can impair DNA damage recognition and repair, contributing to drug resistance, particularly via mechanisms such as miRNA-21 overexpression, which inhibits these mismatch repair (MMR) proteins (Haider et al., 2020). Gene loss due to recurrent chromosomal loss or structural alterations during cellular division, or modifications in metabolic pathways, plays a significant role in triggering resistance to treatments. The TP53 tumor suppressor maintains genome stability and cellular balance by regulating various signaling pathways, and mutations in this gene can lead to drug resistance, invasion, and metastasis (Bukowski et al., 2020). Deficiencies in the mismatch repair (MMR) pathway significantly contribute to drug resistance in tumor cells. For instance, tumors characterized by MMR deficiencies initially exhibit a degree of responsiveness to temozolomide (TMZ) but subsequently develop resistance (Yamashiro et al., 2020). The base excision repair pathway, which is responsible for repairing small base lesions, confers resistance to alkylating agents such as temozolomide (TMZ) and cisplatin (Bai et al., 2020). Moreover, members of the phosphatidylinositol 3-kinase-like family of serine/threonine protein kinases (PIKKs), such as ATM and ATR signaling, are vital in regulating repair mechanisms (Kulkarni et al., 2022). DNA damage response (DDR) mechanisms can repair this damage, leading to resistance against cancer therapies (Yu et al., 2024). Additionally, metabolic changes in cancer cells, such as enhanced glycolysis, generate reactive oxygen species (ROS), which damage DNA and activate DDR pathways (Tiek and Cheng, 2022; Das et al., 2023). Gaining insights into DNA repair mechanisms is crucial for managing drug resistance. Approaches such as targeted inhibitors, combination therapies, and personalized medicine can strengthen treatment efficacy.

### 2.6 Tumor microenvironment in cancer drug resistance

Initially, elevated levels of drug efflux transporter proteins, epigenetic abnormalities, apoptosis evasion, and DNA repair mechanisms were recognized as the underlying factors contributing to drug resistance. However, the concept of the tumor microenvironment (TME) was first proposed by Stephen Paget. He drew an analogy between the interaction of breast carcinoma and its microenvironment to the term “seed and soil” (Ni et al., 2021). The TME encompasses a heterogeneous collection of cellular constituents, such as leukocytes, fibroblasts, and endothelial cells, which are present within the neoplasm or in its adjacent milieu. The diverse composition of the TME protects cancer cells from drug treatments either by releasing soluble molecules like cytokines and chemokines or through continuous cell interactions between tumor and non-tumor cells (Santos and Almeida, 2020). Tumor initiation, progression, metastasis, and resistance to therapy are actively promoted by noncancerous cells within the TME. The abnormal conditions within the TME also lead to changes in the surrounding tissue that further promote tumor development and resistance. Certain types of non-cancerous cells in the TME, such as cancer-associated fibroblasts, macrophages, adipocytes, and fibroblasts, can provide a protective environment for tumor cells to evade immune system targeting (P. Wu et al., 2021). Hence, a comprehensive analysis of the TME’s role in drug resistance is vital for effective cancer treatments.

### 2.7 Immunological mechanisms in cancer drug resistance

The emergence of drug resistance in cancer cells is a multifaceted phenomenon associated with multiple immunological mechanisms. These mechanisms are generally classified into intrinsic and acquired resistance due to their complex interaction between tumor cells, the immune system, and the tumor microenvironment. In adaptive mechanisms, tumor cells suppress T-cell activity by increasing immune checkpoints like PD-L1 (Programmed Death-Ligand 1). For example, under hypoxic conditions in glioblastoma, tumor cells resist immunotherapy by actively secreting exosomes containing PD-L1, which inhibit T-cell function (To and Cho, 2022). Tumor cells also confer resistance to immune-mediated destruction by activating pro-survival signaling pathways, such as the PI3K/Akt/mTOR and RAS/RAF/MAPK pathways, which promote cell proliferation and survival while inhibiting apoptosis. Similarly, the Wnt–β-catenin pathway can stimulate stemness and self-renewal in tumor cells, thereby diminishing their susceptibility to therapeutic interventions (Antoun and Chioni, 2023). Moreover, chronic immune activation occurs due to prolonged exposure to antigens, leading to the secretion of immunosuppressive cytokines like TGF-β and IL-10 (Aktar et al., 2022). Therefore, a comprehensive understanding of these mechanisms will aid in the development of personalized anticancer therapies to combat drug resistance.

### 2.8 Enzymatic mechanisms in cancer drug resistance

Enzymatic mechanisms play a pivotal role in cancer drug resistance by altering drug metabolism, activation, and detoxification. Overexpression of CYP3A4 in hepatocellular carcinoma induces metabolic resistance to docetaxel by suppressing its inhibitory role (Hofman et al., 2021). Phase II metabolic enzymes, such as glutathione S-transferases (GSTs) and UDP-glucuronosyltransferases (UGTs), contribute to drug resistance by conjugating chemotherapeutics for elimination. For instance, upregulation of GSTP1 and GSTA2 leads to chemoresistance in triple-negative breast cancer and other cancers, while UGT1A and UGT2B7 confer resistance against methotrexate and epirubicin. The pregnane X receptor (PXR) may regulate GST and UGT expression, potentially playing a role in TNBC drug resistance. However, further research is needed to confirm this mechanism (Rao et al., 2023). Superoxide dismutase 3 (SOD3), an antioxidant enzyme, is highly expressed in lung cancer and is associated with worse survival outcomes, suggesting the exploration of the role of SOD3 in chemotherapy resistance (Saxena et al., 2022; Zhang et al., 2022). Another enzyme, aldehyde dehydrogenase (ALDH), could be a potential target to improve treatment outcomes in various cancers due to its role in chemotherapy resistance by detoxifying toxic aldehydes, preventing oxidative stress, and inhibiting DNA damage-induced cell death (Zanoni et al., 2022; Al-Shamma et al., 2023). Hence, the roles of CYP3A4, GSTs, UGTs, SOD3, and ALDH highlight the complexity of metabolic resistance in various cancers. Targeting these enzymes through novel inhibitors or combination therapies may enhance treatment efficacy and overcome resistance.

## 3 Nanomaterials in cancer therapy

**FIGURE 3:**
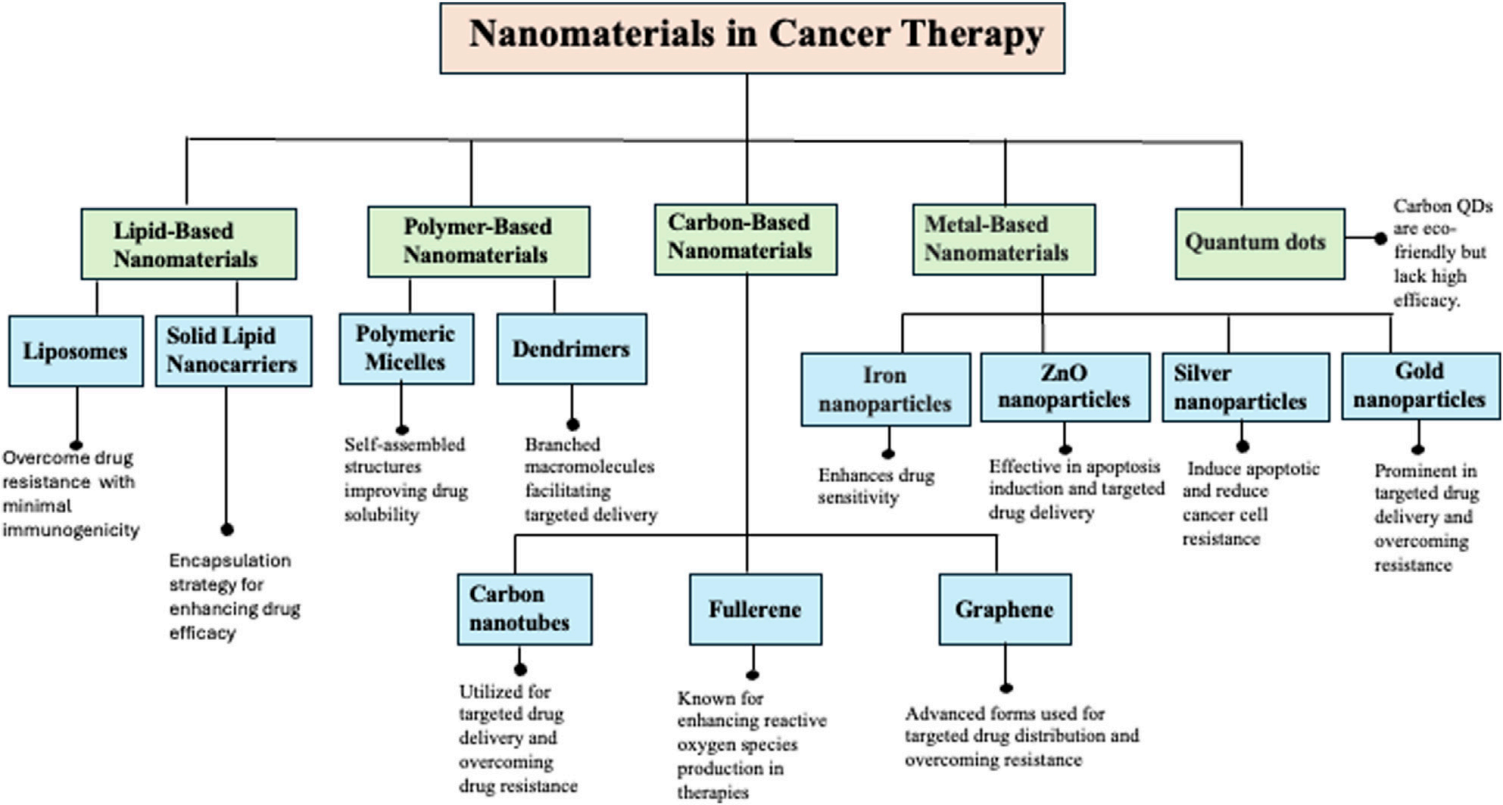
Illustration of the diverse classes of nanomaterials (NMs) used in cancer therapy, highlighting their specific types (e.g., liposomes, dendrimers, metallic nanoparticles, polymeric NPs), primary biomedical applications, and mechanisms of action. These include enhancing targeted drug delivery, improving drug solubility and bioavailability, and overcoming multidrug resistance in cancer cells.

Nanotechnology is a cutting-edge approach in medicine due to the unique properties and diverse applications of nanomaterials (NMs). NMs are ultrafine particles composed of atoms, molecules, or ions ranging from 1 to 100 nm (Ray et al., 2021). Due to their exceptional physicochemical characteristics, such as small size, high biocompatibility, and large surface area, NMs are engineered to perform various functions effectively (Jin et al., 2020). However, delivering nanoparticles (NPs) to the tumor microenvironment (TME) requires overcoming multiple physiological and biological barriers, such as the epithelium, endothelium, and cellular membranes. Therefore, it is imperative to implement precise and enhanced strategies for targeted cellular and nuclear therapeutics (Gavas et al., 2021). To address these challenges, a variety of nanomaterials, including lipid-based NMs, polymer-based NMs, carbon-based NMs, metal-based NMs, and quantum dots, have been developed to combat drug resistance in cancer therapy (Figure 3).

### 3.1 Lipid-based nanomaterials

Lipid nanoparticles have emerged as highly biocompatible carriers since the 1990s and are recognized as “nano-safe” for efficient drug delivery (Sheoran et al., 2022). In 2021, Majumder and Minko. (2021) designed lipid-based NMs for the precise delivery of luteinizing hormone-releasing hormone (LHRH) to non-small cell lung cancer, using LHRH ligands. Their findings demonstrated inhibition of EGFR signaling and an enhanced anticancer effect through the co-delivery of paclitaxel with siRNA. Types of lipid-based NMs include liposomes, nanoemulsions (NEs), solid lipid nanocarriers (SLNs), nanostructured lipid carriers (NLCs), lipid-polymer hybrid nanoparticles (LPH-NPs), and exosomes. These lipids, when used in the creation of lipid nanoparticles (LNPs), are non-toxic, compatible with biological systems, and biodegradable, exhibiting minimal to no immunogenicity (Chaudhuri et al., 2022). Lipid-based NMs have significant potential to overcome drug resistance by enhancing drug solubility, bioavailability, and controlled release. By modifying surface properties and integrating targeted ligands, these nanocarriers improve drug retention, site-specific delivery, and therapeutic efficacy in resistant cancer cells.

#### 3.1.1 Liposomes

Nanotechnology is a cutting-edge tool in medicine due to the unique properties and applications of nanomaterials (NMs). These ultrafine particles, ranging from 1 to 100 nm, exhibit exceptional physicochemical properties, including small size, high biocompatibility, and large surface area (Ray et al., 2021). However, delivering nanoparticles (NPs) to the tumor microenvironment (TME) requires overcoming several physiological and biological barriers, such as the epithelium, endothelium, and cellular membranes. Therefore, it is essential to implement advanced strategies for targeted cellular and nuclear therapeutics (Gavas et al., 2021). To overcome these challenges, a variety of nanomaterials, including lipid-based, polymer-based, and carbon-based NMs, have been developed for targeted drug delivery. One such approach involves the use of ginsenoside Rg3-based liposomes (Rg3-PTX-LPs) to counteract paclitaxel (PTX) resistance in breast cancer (Zhu R. et al., 2022; Zhu Y. et al., 2022). These liposomes effectively target taxol-resistant MCF-7 human breast cancer cells (MCF-7/T) through GLUT-1 recognition, reversing drug resistance by inhibiting the IL-6/STAT3 pathway, repolarizing M2 macrophages to M1, and reducing tumor-associated fibroblasts and myeloid-derived suppressor cells. As a result, Rg3-PTX-LPs achieved a 90.3% tumor inhibition rate, demonstrating their potential as a multifunctional strategy for overcoming drug resistance in cancer therapy. However, challenges related to long-term stability, immunogenicity, and clinical feasibility remain to be addressed.

Apolipoprotein A1 (ApoA1)-modified cationic liposomes represent another promising strategy to enhance drug delivery for MDR cancer therapy (An et al., 2021). These liposomes improved doxorubicin (Dox) uptake, reduced off-target effects, and demonstrated superior anti-tumor efficacy compared to free Dox in MCF-7/ADR cells. Additionally, the ApoA1-lip/Dox system minimized adverse effects on cardiac function and other organs, highlighting its potential for overcoming MDR in cancer treatment. While the research demonstrates improved therapeutic efficacy in MDR cancer models, further studies on pharmacokinetics, biodistribution, and potential resistance mechanisms against ApoA1-lip/Dox are essential for validating clinical translation.

Beyond conventional nanotechnology-based drug delivery, a hybrid therapeutic approach integrating nanotechnology with epigenetic modulation is emerging as a promising strategy to overcome resistance mechanisms in cancer. Glycolysis, which is promoted by histone deacetylation, contributes to resistance against tyrosine kinase inhibitors (TKIs) like osimertinib. This challenge can be addressed using a hybrid therapy that involves liposomal codelivery of osimertinib and panobinostat, effectively targeting both tumor cells and the microenvironment to overcome drug resistance via tumor-associated macrophage (TAM) repolarization and glycolysis inhibition (Lin et al., 2023). However, concerns about immunogenicity, rapid clearance upon repeated administration, and off-target effects necessitate further research to develop more precise and biocompatible alternatives for sustained clinical success. By integrating these novel nanotechnology-driven strategies, including targeted liposomal delivery systems and hybrid therapeutic approaches, researchers can advance the fight against MDR in cancer, ultimately improving patient outcomes and therapeutic efficacy.

#### 3.1.2 Solid lipid nanocarriers

Solid Lipid Nanocarriers (SLNs) offer a promising strategy for overcoming drug resistance in cancer cells by encapsulating both hydrophilic and hydrophobic drugs, enhancing solubility, bioavailability, and controlled release. C-peptide-decorated SLNs (C-peptide-SLNs) enhance paclitaxel (PTX) cytotoxicity against resistant 4T1 carcinoma cells, achieving an IC50 of 1.2 µg/mL—lower than free PTX and SLN-PTX—by targeting αvβ3 integrin receptors. *In vivo*, they reduced tumor volume by 82%, prevented pulmonary metastasis, and minimized systemic toxicity, as evidenced by normalized liver enzyme levels. Immunohistochemical analyses further confirmed increased apoptosis and inhibited tumor proliferation, highlighting their potential in triple-negative breast cancer treatment. However, the potential off-target effects and receptor heterogeneity need to be considered to optimize therapeutic specificity (Rahdari et al., 2025). Further research is required to assess long-term stability, biodistribution, and potential immune responses to validate effective cancer therapy against resistant cancer cells. In another approach, Darabi et al. (2022) demonstrated that SLNs enable a controlled and sustained release of doxorubicin, with only 6.5% released in the first hour but reaching 96.16% after 48 h, ensuring prolonged drug availability in resistant cancer cells. Functionalizing SLNs with dual aptamers (SLNs/DOX/Dexa/CD44/EGFR) significantly enhanced cytotoxicity compared to non-targeted SLNs, reinforcing the advantage of multi-pathway targeting in overcoming drug resistance. These findings suggest that dual-aptamer-functionalized SLNs represent a promising strategy for improving drug delivery and therapeutic efficacy in resistant cancer treatment (Darabi et al., 2022). Despite these advancements, SLNs face challenges such as high moisture content, poor drug loading, and stability issues during storage. To address these limitations, researchers have developed a second-generation lipid nanoparticle system known as Nanostructured Lipid Carriers (NLCs), which provide improved stability and enhanced drug-loading capacity. Notably, formulations such as Nano Repair Q10 cream and serum have demonstrated the practical applications of NLCs in targeted drug delivery (Sheoran et al., 2022).

### 3.2 Polymer-based nanomaterials

Polymer-based NMs play a significant role in drug resistance in cancerous cells due to their nominal cytotoxicity, enhanced solubility, versatility, and mechanical robustness. Their distinctive characteristics make them ideal for transporting a broad range of drugs to the targeted site to combat drug resistance in cancerous cells (Salari et al., 2022). Studies have shown that polymeric NMs are pivotal in cancer detection and management, personalized therapies, and regenerative medicine. Based on their specific properties, these NMs, including polymeric micelles and dendrimers, can be useful in defeating various types of cancers (Idrees et al., 2020).

#### 3.2.1 Polymeric micelles

Polymeric micelles are self-assembled, biphasic structures consisting of a hydrophilic outer shell and a hydrophobic inner core, making them highly effective carriers for drug delivery. In aqueous solutions, they form stable structures composed of amphiphilic block copolymers, with core-forming components such as polyesters, polyethers, and polyamino acids, while hydrophilic polymers like PEG, chitosan, dextran, and hyaluronic acid enhance their stability and functionality (Ghosh and Biswas, 2021). Due to their unique architecture, polymeric micelles play a crucial role in targeted drug delivery for resistant tumor cells by improving drug solubility, retention, and minimizing systemic side effects. One of their key mechanisms in cancer therapy is the exploitation of the Warburg effect, where cancer cells rely on glycolysis for energy. By capturing glucose within the tumor microenvironment, these micelles disrupt glucose metabolism, inducing energy stress and apoptosis in cancer cells. Unlike conventional anticancer drugs, which often encounter resistance, polymeric micelles offer a novel approach by directly intensifying metabolic stress rather than relying on cytotoxic agents. Their versatility allows them to encapsulate both hydrophilic and hydrophobic drugs, enhancing therapeutic efficacy while ensuring targeted tumor accumulation through renal clearance, thereby reducing systemic toxicity.

Lee et al. (2022) these micelles have shown particular effectiveness in delivering resveratrol, significantly increasing its uptake in breast cancer cell lines (MDA-MB-231 and MCF-7) while sparing normal cells. This selective action may be attributed to modifications in membrane microviscosity induced by the block copolymers, facilitating greater drug absorption. Additionally, the incorporation of Vitamin E TPGS stabilizes the formulation while enhancing proapoptotic effects, further improving anticancer efficacy (Gregoriou et al., 2020). While these approaches offer a promising strategy to overcome drug resistance, its long-term impact on normal glucose metabolism in non-cancerous tissues requires further investigation. Moreover, the ability to integrate real-time monitoring into these micelles presents an opportunity for improved theragnosis, but challenges in clinical translation, including scalability and regulatory approval, must be addressed.

#### 3.2.2 Dendrimers

Dendrimers are branched macromolecules with a core-shell structure, ranging from 1 to 15 nm in size. Each successive branching layer, known as a “generation,” increases molecular weight and functionality, making dendrimers highly versatile for biomedical applications. Their distinctive 3D architecture enables them to serve as efficient drug-delivery agents, addressing drug resistance, and as detectors for ions or biomolecules (Ray et al., 2021). Commercially available dendrimers, such as polyamidoamine (PAMAM) and polylysine, have been widely used in drug-resistant cancer cells to enhance treatment efficacy (Gavas et al., 2021). One of the key applications of dendrimers in cancer therapy is their ability to facilitate targeted drug delivery. Through antibody modifications like 2C5, they improve drug accumulation at tumor sites while minimizing off-target effects. Additionally, dendrimers enable the co-delivery of small interfering RNA (siRNA) and chemotherapeutic agents, with siRNA downregulating P-glycoprotein (P-gp), an efflux pump responsible for drug resistance. This mechanism increases intracellular drug concentration, making chemotherapy more effective against MDR cancer cells. Furthermore, dendrimer-based formulations are biocompatible, non-hemolytic, and exhibit long-term stability, ensuring their safety and effectiveness in treating cancers such as triple-negative breast cancer and ovarian cancer (Yalamarty et al., 2022). Among different dendrimer types, polyamidoamine PAMAM dendrimers stand out as particularly effective drug carriers due to their highly branched structure, which enables precise drug encapsulation and delivery. Their surface charge significantly influences cellular uptake and exocytosis, with positively charged PAMAM-NH2 dendrimers exhibiting the highest exocytosis rates in resistant cancer cells, while neutral PAMAM-OH dendrimers have the lowest. Moreover, PAMAM dendrimers can help overcome MDR by delivering agents that downregulate MDR genes, restoring drug sensitivity in resistant tumors (Zhang et al., 2021). However, despite their advantages, the surface charge-dependent interactions of dendrimers can also lead to toxicity, making it essential to balance therapeutic efficacy with biocompatibility for safe clinical applications.

### 3.3 Carbon-based nanomaterials

Carbon, due to its versatile electron configuration and hybridization, plays a crucial role in DNA structure. Carbon-based nanomaterials (CBNs), including nanotubes, fullerenes, and graphene, have advanced targeted drug delivery and nanotherapeutics, thanks to their ability to carry large amounts of drugs, distinctive optical properties, and high biocompatibility. However, challenges such as toxicity and bio-corona formation need to be addressed to enhance their biomedical applications for cancer treatment (Debnath and Srivastava, 2021; Hosseini et al., 2023).

#### 3.3.1 Carbon nanotubes

Carbon nanomaterials leverage their optical, mechanical, and electronic properties, along with their biocompatibility, to combat drug resistance in cancer treatment. Research shows that carbon nanotubes (CNTs) are efficiently internalized by various cell types due to their needle-like structure, making them effective drug carriers. By delivering therapeutic molecules directly to key intracellular sites, such as the nucleus and mitochondria, CNTs help disrupt the tumor microenvironment (TME) and interfere with cancer cell survival mechanisms (Tang et al., 2021).

A promising advancement in this field is magnetically controlled carbon nanotubes (mCNTs), which use rotational magnetic fields to mechanically disrupt drug-resistant glioblastoma (GBM) cells, leading to cell death. Unlike traditional chemotherapy, which targets biochemical pathways, mCNTs use physical force to eliminate resistant GBM cells. Functionalization with anti-CD44 antibodies further enhances their tumor targeting and retention, improving treatment efficacy. Additionally, the biocompatible carbon surface of mCNTs minimizes toxicity, ensuring safe application in preclinical GBM models (Wang et al., 2023).

Multiwalled carbon nanotubes (MWCNTs) have also shown promise as effective drug carriers, particularly for chemotherapeutic agents like cisplatin. In cisplatin-resistant non-small cell lung cancer (NSCLC) cells, cisplatin-loaded MWCNTs (Pt-MWCNTs) reversed resistance by regulating the epithelial-mesenchymal transition (EMT)—a key process in cancer metastasis. This led to a significant reduction in cancer cell invasiveness while promoting apoptosis. *In vivo* studies further demonstrated that Pt-MWCNTs inhibited tumor growth more effectively than free cisplatin, highlighting their potential to enhance chemotherapy efficacy (Qi et al., 2021).

However, further research is required to evaluate long-term immune interactions, which may impact clinical safety and effectiveness. Additionally, CNTs enhance targeted drug delivery by efficiently penetrating cell membranes without causing immediate toxicity, allowing for higher drug loads and improved therapeutic effects. Functionalized multi-walled carbon nanotubes conjugated with bromocriptine (BRC) specifically target cancer cell receptors, significantly increasing drug internalization and cytotoxicity in resistant lung cancer cells while sparing normal cells. This study demonstrated that MWCNTs-BRC significantly lowered IC50 values, underscoring their ability to overcome drug resistance (Kamazani et al., 2021).

While CNT-based drug delivery holds great promise, several challenges remain. Large-scale production, long-term biodistribution, and potential immune responses must be carefully assessed before these technologies can transition into clinical applications. Furthermore, the variability in CNT formulations may influence their toxicity profiles, necessitating further investigation into optimizing their biocompatibility and regulatory approval.

#### 3.3.2 Fullerene

Buckminsterfullerene (C60) is a spherical fullerene discovered in 1985 that shows promise as a photosensitizer for producing singlet oxygen, making it valuable in applications like photodynamic cancer therapy and blood sterilization. Fullerenes can enhance targeted therapies and overcome drug resistance by increasing reactive oxygen species (ROS) production due to their antioxidative properties. However, the clinical advancement of fullerene-based nanomedicine is limited by concerns over long-term safety and toxicity (Farani et al., 2024).

A recent study by Fawal et al. (2023) highlighted the potential of functionalized fullerenes as powerful anticancer agents, particularly against resistant cancer cells. The Fullerene@CA nanocomposite fibers significantly inhibited cancer cell growth and induced apoptosis, with fullerene-loaded fibers showing higher apoptotic activity than free fullerene. This study emphasized fullerene’s ability to stimulate the MEK-ERK pathway and selectively target resistant cancer cells while sparing normal cells. However, further research is needed to assess its long-term biocompatibility and potential systemic effects in clinical applications.

Similarly, C60 fullerene derivatives show significant promise in overcoming cancer drug resistance through various mechanisms for enhanced therapeutic efficacy. A study by Horak et al. (2021) explored how C60 fullerene acts as a nanoplatform for non-covalent drug complexation, improving the intracellular uptake of drugs like Berberine, Doxorubicin, and Cisplatin, thus enhancing their cytotoxic effects at lower concentrations. This strategy not only reduces cell proliferation and metastasis but also reverses cancer cell invasiveness by modulating EMT markers and stimulating immune responses in the TME. Additionally, Fu et al. (2024) demonstrated that C60 photosensitized drug delivery systems could effectively improve anti-tumor efficacy and reduce toxicity in colorectal cancer treatments. C60 nanoparticles generate ROS upon laser activation to induce apoptosis, with functionalization improving solubility and targeted delivery. Targeting the FOLR1 protein on cancer cells ensures selective toxicity, reducing side effects. Despite the promising potential of fullerene-based therapies, both studies emphasize that the long-term safety, stability, and potential off-target effects of C60 derivatives in clinical applications remain uncertain, necessitating further research to fully evaluate their clinical potential. These findings provide valuable insights into the potential of fullerenes as targeted drug delivery systems for overcoming drug resistance in cancer cells. However, challenges remain in achieving consistent fullerene association with certain chemicals, limiting their universal application as an effective drug delivery system.

#### 3.3.3 Graphene

Graphene, a monolayer carbon nanomaterial discovered in 2004, has demonstrated the ability to cross biological barriers and interact with cellular components, though its potential cytotoxicity and DNA fragmentation remain concerns. To enhance its biomedical applications, advanced forms such as graphene oxide (GO) and graphene quantum dots have been developed, offering improved targeting capabilities and immune response activation to combat drug resistance in cancer cells (Dilenko et al., 2024). Graphene and GO exhibit key properties like high biocompatibility, targeted drug distribution, and the ability to induce ROS, which are crucial for overcoming chemotherapy resistance.

A study by Yaghoubi et al. (2022) investigates the application of functionalized GO in co-delivering doxorubicin (DOX) and curcumin (CUR) for cancer therapy. Their findings highlight GO’s effectiveness as a nanocarrier, significantly enhancing drug bioavailability while minimizing side effects. Notably, GO-COOH functionalization increases the cytotoxic impact on human gastric (AGS), prostate (PC3), and ovarian (A2780) cancer cell lines. The study also emphasizes the pH-sensitive drug release mechanism, where drugs are optimally released in the acidic tumor microenvironment, maximizing therapeutic efficacy while reducing toxicity to normal cells.

Building on these advancements, Guo et al. (2021) developed a novel drug delivery system utilizing GO-based nanosheets modified with polyethylene glycol (PEG) and oxidized sodium alginate (OSA), termed PTX@GO-PEG-OSA. This system employs a pH/thermal-sensitive mechanism to release paclitaxel (PTX) in gastric cancer (GC) treatment, particularly targeting drug-resistant cells. The application of near-infrared (NIR) irradiation further enhances therapeutic effects by generating ROS, disrupting mitochondrial function, and reducing ATP production, which weakens the P-glycoprotein (P-gp) efflux pump responsible for drug resistance. Consequently, this treatment demonstrates significantly improved antitumor efficacy compared to conventional chemotherapy. These studies underscore the potential of graphene-based nanocarriers in developing advanced cancer treatments. However, while GO and its derivatives offer promising strategies for overcoming drug resistance, challenges such as cytotoxicity, long-term biocompatibility, and interactions with biological molecules must be addressed before clinical implementation.

### 3.4 Metal-based nanomaterials

Metal-based nanoparticles (NPs), such as iron, ZnO, gold, and silver, have demonstrated remarkable potential in cancer therapy. Their distinctive surface chemistry, nanoscale size, and physicochemical properties contribute to applications in imaging, protein interactions, DNA hybridization detection, and photothermal therapy. Notably, these properties also play a critical role in overcoming drug resistance in cancer cells (Kumar et al., 2023; Chandrakala et al., 2022). Among these, gold and silver nanoparticles have shown exceptional promise due to their efficacy in preclinical studies. Their cost-effectiveness and multifunctionality make them viable alternatives to conventional treatments for drug-resistant cancers (Hheidari et al., 2024).

#### 3.4.1 Iron nanoparticles

Iron nanoparticles have gained attention for their superior reactivity and ability to act as remediation agents compared to conventional iron powders (Xu et al., 2021). Iron oxide nanoparticles, in particular, can induce ferroptosis—a regulated form of cell death driven by iron-mediated lipid peroxidation. This mechanism has shown significant efficacy in targeting drug-resistant cancer cells that evade apoptosis (Sheikh et al., 2024). A key advantage of iron oxide nanoparticles is their ability to be guided to tumor sites using external magnetic fields, minimizing off-target effects and improving bioavailability. For instance, magnetic Fe_3_O_4_ nanoparticles have been shown to enhance drug sensitivity in multidrug-resistant liver cancer cells. This effect was demonstrated by a significant reduction in the half-maximal inhibitory concentration of chemotherapeutic agents such as ADM, DDP, 5-FU, and VCR, alongside increased programmed cell death and decreased expression of resistance-associated proteins (STAT3 and survivin) (Chen et al., 2022).

Furthermore, iron nanoparticles can be engineered to respond to various therapeutic modalities. For example, Fe^3+^ ion-chelated PDA nanoparticles leverage the Fenton reaction to enhance ferroptosis under near-infrared light exposure. Similarly, microwave-assisted thermal therapy has shown promise, with copper-cysteamine nanoparticles effectively generating reactive oxygen species (ROS) and inducing lipid peroxidation under microwave exposure. Radiotherapy, another modality, utilizes ionizing radiation to generate ROS, while hybrid nanoplatforms such as HMON-GOx@MnO_2_ have demonstrated strong ferroptosis-inducing effects. Additionally, sonodynamic therapy, when combined with ferroptosis inducers, has exhibited promising outcomes, including IRP NPs targeting mitochondria to trigger ferroptosis in triple-negative breast cancer cells (Zhu R. et al., 2022; Adzavon et al., 2024). Iron-based nanomaterials continue to evolve, with graphene-nanochainmail-protected catalysts offering enhanced stability and efficiency to address durability challenges in catalytic processes. In the context of drug resistance, engineered iron nanoparticles hold great potential for improving drug delivery, modulating the tumor microenvironment, and overcoming resistance mechanisms, ultimately leading to more effective therapeutic strategies (Zhang et al., 2022). Despite these advancements, challenges remain in translating these findings into clinical applications. Factors such as long-term toxicity, stability, and regulatory approval must be addressed to ensure their safety and efficacy. Ongoing research is crucial to refining these techniques and optimizing their clinical viability.

#### 3.4.2 ZnO nanoparticles

Zinc oxide nanoparticles (ZnO-NPs) have demonstrated significant antitumor activity in various cancers, including breast, liver, lung, and colon cancer, by inducing apoptosis through oxidative and proteotoxic stress. Additionally, ZnO-NPs enhance the pro-apoptotic effects of chemotherapy drugs like cisplatin and gemcitabine in non-small cell lung cancer. However, their role in overcoming ovarian cancer resistance remains unclear (Gu and Yang, 2023). Zhou et al. engineered a ZnO-based nanomedicine platform that responds to both elevated matrix metalloproteinase-2 levels and acidic tumor pH, enabling targeted delivery of doxorubicin in multidrug-resistant cancer cells. The ZnO hybrid formulation facilitated enhanced cellular internalization, tumor-specific targeting, and improved anticancer activity against MDR tumors, while concomitantly reducing toxicity compared to free doxorubicin and non-responsive ZnO nanoparticles (Zhou et al., 2023). Thakral et al. investigated the antiproliferative effects of ZnO nanoparticles synthesized from soybean seed extract, which exhibited dose-dependent cytotoxicity and enhanced anticancer potential when combined with levofloxacin, highlighting their promise for future therapeutic applications (Thakral et al., 2023). However, Wu et al. (2022) discovered the potential resistance of ZnO nanoparticles to chemotherapeutic drugs in colon cancer cells through oxidative stress and Nrf2 activation. The findings suggest that ZnO NPs, particularly those with pristine, NH2-, and COOH-functionalized surfaces, promote a cytoprotective response that enhances drug resistance, whereas silica-coated ZnO NPs have negligible effects. This study highlights the need for further research on engineered nanomaterial-induced drug resistance using *in vivo* models and for examining ENM–drug interactions in chemotherapy disruption. Understanding the impact of ENM exposure could help assess the disproportionate risks for certain populations and inform safer nanomaterial design strategies (Wu et al., 2022).

#### 3.4.3 Silver nanoparticles

Silver nanoparticles (AgNPs) stand out for their peculiar chemical, optical, and biological properties, and are extensively used in anticancer therapeutics aimed at tackling drug resistance in cancerous cells (Gomes et al., 2021; Swilam and Nematallah, 2020; Qin et al., 2021; Hasanin et al., 2021). In Oves et al. (2022) investigated the apoptotic potential of AgNPs through Hoechst staining, showing DNA condensation and apoptotic bodies in treated samples compared to healthy cells. The study disclosed that the cytotoxic effects of AgNPs on MDA MB-231 cells vary with concentration and time, displaying an IC50 value of 16.8 μg/mL at 24 h with minimal cell viability at higher concentrations. Biocompatibility assays, utilizing the lysis of normal erythrocytes, demonstrated that biologically synthesized AgNPs exhibit negligible cytotoxicity, even at a concentration of 50 μg/mL on hematological cells (Oves et al., 2022). These findings suggest that biologically synthesized AgNPs possess efficacy in activating apoptosis in tumor cells and exhibit minimal toxicity toward normal erythrocytes, which may help minimize drug resistance in cancer cells.

#### 3.4.4 Gold nanoparticles

Gold nanoparticles are garnering more attention from researchers for targeted drug delivery systems due to their elevated uptake by cells, biocompatibility, hydrophilicity, non-immunogenicity, and minimal toxicity, which help address drug resistance in cancerous cells (Soliman et al., 2023; Pasparakis, 2022). Moreover, several ligands can be immobilized onto gold nanoparticles for modulated drug release (Yafout et al., 2021). In a study by Perveen et al. (2021), synthesized gold nanoparticles using *Trachyspermum ammi* seed extract demonstrated anticancer potential against HepG2 cancer cells by triggering ROS-mediated apoptosis. Another group of researchers, Y. Yu et al. (2022), conducted research using temozolomide (TMZ)-loaded gold nanoparticles (anti-EphA3-TMZ@GNPs) to reduce glioblastoma (GBM) resistance to TMZ and enhance therapeutic efficiency. They observed significant cellular uptake, activated apoptosis, and overcame drug resistance. The incorporation of these nanoparticles with plasma photothermal treatment displayed significant cellular uptake, activated apoptosis, and nullified drug resistance by regulating apoptotic signaling and minimizing MGMT expression, a DNA repair enzyme associated with resistance to TMZ. *In vivo* studies emphasize this method as a promising treatment strategy for GBM due to extended survival, efficient brain penetration, and safety of these synthesized gold nanoparticles (Y. Yu et al., 2022). In conclusion, gold nanoparticles, with their unique properties and ability to enhance targeted drug delivery, show significant promise in overcoming drug resistance in cancer cells, offering a potential strategy to improve the efficacy of cancer therapies.

### 3.5 Quantum dots

Quantum dots (QDs) are a class of nanomaterials characterized by their semiconducting properties and distinctive optical and electrical characteristics, which may be effective in combating drug resistance in cancer cells by producing ROS and facilitating the conjugation of pharmaceutical agents, antibodies, and adjuvants. Graphene-based QDs demonstrate effectiveness in addressing MDR through the negative regulation of MDR-related genes and enhancing drug retention in cancer cells (Yadav at al., 2022). Moreover, carbon quantum dots have the potential for controllable and reproducible industrial production due to their eco-friendly characteristics and economical, comparatively simpler manufacturing process. Unfortunately, CQDs exhibit concentration-dependent toxicity, imposing restrictions on their potential implementation. Therefore, it is crucial to address the challenge of toxicity and assess the precision of cancer cell targeting in carbon-based therapies.

## 4 Enhanced permeability and retention (EPR) effect

The EPR effect is a powerful passive targeting mechanism that utilizes the unique features of the tumor vascular system to deliver drugs directly to malignant cells. This targeted strategy ensures that pharmacological agents are primarily confined within the neoplasm, thereby accelerating therapeutic effectiveness while simultaneously reducing adverse effects on surrounding healthy tissues (Mandal, 2023). Moreover, a study by Sell et al. (2023) described how the functionalization of NMs with specific targeting ligands enhances their ability to transport drugs directly to neoplastic targets. They observed a significant reduction in side effects and increased treatment efficacy, representing a remarkable advancement in therapeutics. Ma et al. (2024) employed an innovative approach to demonstrate the enhanced EPR effect and therapeutic efficacy in controlling invasive pituitary adenomas, showcasing the potential for clinical translation. They highlighted Folliculostellate Cell Membrane-Coated Nanoparticles (FSNPs), which, due to their inheritance of membrane proteins from folliculostellate cells, may promote improved interactions with the tumor microenvironment (TME). This facilitates the accumulation of nanoparticles at the tumor site via the EPR effect. The ability of FSNPs to mimic the natural cell membrane enables them to evade the immune system more effectively than standard nanoparticles, which is crucial for maximizing the EPR effect. The study indicates that the encapsulation of mitotane within FSNPs significantly enhances therapeutic efficacy in pituitary adenoma models, suggesting that the improved EPR effect directly contributes to better drug delivery and effectiveness.

Recently, Yu et al. (2024) combined MUC18-targeted gold nanorods with mild hyperthermia to increase tumor endothelial permeability. MUC18 is a cell surface glycoprotein that plays a critical role in cellular adhesion and signaling, extending its involvement to various processes such as migration and proliferation. They observed extensive intercellular gaps in the tumor endothelium caused by the gold nanorods, alongside mild hyperthermia, which induced actin remodeling and cell contraction. Since MUC18 is overexpressed in various tumors, this approach offers a promising method to enhance cancer therapy by improving tumor endothelial permeability.

## 5 Nanoparticle-based cancer immunotherapy

Encapsulation of anticancer agents using nanoparticles enhances drug accumulation at tumor sites, prolongs circulation, and improves treatment efficacy. Polymer-based antibody-conjugated nanoparticles leverage the enhanced permeability and retention (EPR) effect, enabling selective drug delivery while minimizing toxicity. Monoclonal antibody (mAb)-based drug delivery, particularly trastuzumab (Tmab), has significantly advanced breast cancer (BC) therapy by targeting HER2-overexpressing tumors. Polymeric nanoparticles (PNPs) and liposomal carriers provide promising alternatives by improving drug stability, targeted delivery, and minimizing side effects. These strategies can enhance the efficacy of Tmab while making treatment more accessible in resource-limited settings (Selepe et al., 2024). Furthermore, nanoparticle-based drug delivery systems (NDDS) trigger tumor-specific immune activation by releasing damage-associated molecular patterns to induce immunogenic cell death (Zhou et al., 2022). Moreover, gene editing strategies based on NDDS, such as CRISPR/Cas9-mediated PD-L1 knockout, play a significant role in reversing immunosuppressive mechanisms to elevate anti-tumor immunity (Wang et al., 2021). Additionally, NDDS utilizes manganese dioxide-based nanomaterials to boost oxygen levels, overcoming tumor hypoxia and acidity (Liu et al., 2022). Hydrogels and microneedle patches are also used in nanoparticle-based immunotherapy for controlled and localized drug delivery. For example, ROS-responsive hydrogels loaded with gemcitabine and anti-PD-1 antibodies enable prolonged antibody release and effective tumor treatment, while fibrin gel loaded with anti-CD47 antibodies encapsulated in calcium carbonate nanoparticles facilitates macrophage-mediated tumor cell removal. Additionally, microneedle patches consisting of hyaluronic acid and pH-sensitive dextran nanoparticles trigger immune responses against melanoma by delivering anti-PD-1 antibodies transdermally (Chen et al., 2022). NDDS optimizes cancer treatment and reduces side effects by precisely delivering monoclonal antibodies and immunomodulatory agents. However, sophisticated exploration of NDDS is essential for the clinical potential of monoclonal antibody-based cancer therapy.

### 5.1 Strategies to combat drug resistance using nanoparticles

#### 5.1.1 Targeted drug delivery

Drug resistance remains a significant obstacle in optimizing cancer therapy. Nanoparticles (NPs) offer an advanced solution for enhancing drug delivery by selectively targeting neoplastic cells. These NPs can be engineered to exploit the enhanced permeability and retention (EPR) effect, ensuring a more effective accumulation of drugs in tumor tissue. For instance, a study by Dechbumroong et al. (2024) highlighted the potential of NPs in improving therapeutic outcomes by specifically targeting cancer cells. A significant mechanism underlying drug resistance is the role of Multidrug Resistance Protein-2 (MRP2), which is regulated by hypoxia-inducible factor-1 (HIF-1) and influences the glutathione binding of platinum-based drugs like cisplatin. In a study by Zhang et al. (2020), a microporous silica-based co-delivery system (PMONA) was developed to enhance cisplatin efficacy by co-delivering an HIF-1 inhibitor and a small peptide that binds to platinum. This approach significantly reduced tumor growth by downregulating HIF-1-related proteins, demonstrating the potential of NP systems in enhancing targeted drug delivery.

#### 5.1.2 Combination therapies

Nanoparticles are also increasingly used in combination therapies, where they can co-deliver multiple therapeutic agents to overcome drug resistance. An example of this is found in the work by Yu et al. (2019), where a smart nanoparticle system was used to reduce resistance to cisplatin by incorporating conjugated polymers activated by near-infrared light to generate hyperthermia at 43°C. The design of a novel “shell-core” nanosystem, DOX-PLGA/CPT/PD, successfully addressed drug resistance in MCF-7/ADR breast cancer cells by improving the delivery of doxorubicin into mitochondria. This system involved positively charged lipid-polymer nanoparticles, surrounded by a negatively charged shell, which responded to an acidic environment, leading to successful lysosomal escape and enhanced mitochondrial targeting. The treatment resulted in significant cytotoxicity and a tumor inhibition rate of 84.9%, indicating the efficacy of nanoparticle-based combination therapies in overcoming resistance.

#### 5.1.3 Stimuli-responsive nanoparticles

Stimuli-responsive nanoparticles (NPs) offer innovative solutions for overcoming drug resistance by releasing their payloads in response to specific tumor microenvironment conditions, such as pH. One challenge in targeted therapy arises from gene mutations, efflux pumps, and autophagy, which contribute to resistance after prolonged drug use. For example, AZD9291, a third-generation EGFR tyrosine kinase inhibitor used in non-small cell lung cancer, faces resistance due to mutations like T790M. Gu et al. (2020) explored dual inhibition of EGFR1 and autophagy using pH-sensitive NPs, which enhanced tumor therapy by ensuring lysosomal escape and sequential drug release. The study demonstrated that nanoparticle-based strategies could bypass drug resistance mechanisms by promoting targeted drug delivery through both EPR and active targeting. In a similar vein, Yoo et al. (2024) underscored the potential of co-encapsulating anticancer drugs with resistance inhibitors, improving pharmacokinetic and pharmacodynamic properties while addressing overexpression of resistance-related proteins such as Bcl-2, Akt, and P-glycoprotein (P-gp). By enhancing drug solubility and stability, NP systems can significantly improve therapeutic efficacy while minimizing systemic toxicity, presenting a promising approach to combat drug resistance.

## 6 Challenges and limitations

Despite the progress achieved, the complex nature of NMs and their functional integration poses substantial challenges. European agencies, such as the European Research Council and EURO-NanoTox, emphasize the importance of nanospecific safety and efficacy measurements. Based on morphology and reactivity, precise testing techniques such as Dynamic Light Scattering and Scanning Electron Microscopy could play an important role in determining the interaction of NMs with biological systems in the human body. Nevertheless, advanced research is required due to the ambiguity surrounding the exact mechanism of interaction and the absence of standardized assessment methodologies (Zielińska et al., 2020). Foulkes et al. (2020) discuss the complications in evaluating the safety and efficacy of nanoparticles due to their unique properties. These issues include inconsistent classification across regions and difficulties in scaling up manufacturing while maintaining quality, which remains a significant barrier to large-scale delivery. Furthermore, inadequate expertise, unclear guidelines, and the intricacy of nanomedicine-specific features hamper the advancement and safe inclusion of nanomedicines into clinical use. Moreover, without extensive *in vivo* and long-term studies, the potential adverse effects of prolonged nanoparticle exposure remain uncertain. It has also been noted that the agglomeration of NMs in the liver presents a significant obstacle, as it retains most of the injected dose, reducing bioavailability while increasing immunogenicity and toxicity (Sell et al., 2023). Garriga et al. (2020) conducted a cytotoxicity study to reveal the *in vitro* toxicity of various carbon NMs in human epithelial colorectal adenocarcinoma cells and human breast adenocarcinoma cells. They concluded that ongoing toxicity concerns and contingent cytotoxicity remain critical challenges for clinical applications due to the pronounced hydrophobicity and intricate morphology of carbon NMs. The lack of long-term toxicity and safety data raises serious concerns, as there is currently limited clinical trial research focusing on nanoparticle-based approaches to combat cancer drug resistance. This gap in research not only hampers our understanding of the potential risks associated with these novel therapies but also underscores the urgent need for comprehensive studies to evaluate their efficacy and safety over extended periods. Consequently, exhaustive preclinical and clinical analysis remains vital to gauge the safety and efficacy of cancer treatment utilizing NMs.

## 7 Future directions

Despite significant advancements, challenges remain in addressing the long-term nanotoxicity and pharmacokinetics of carbon NMs. Additionally, standardizing these techniques is essential for overcoming obstacles in their clinical application (Farani et al., 2024). Composite nanocarriers, such as gold-quantum dots, offer complementary cancer treatments due to their versatile potential. These include applications in fluorescence, photothermal therapy, and magnetic resonance imaging (Kashyap et al., 2023). Escaping endosomes to enter the cytoplasm of cancer cells is a major failure for most drugs; however, nanodiamonds, due to their sharp edges, positive charge, and sustainability under high-pressure, high-temperature conditions, tackle this issue effectively. Nevertheless, additional research is needed to standardize comparisons and generalize the use of nanodiamonds due to their inconsistent characterization and reporting methods. Wang et al. (2023) explored the potential of exosomes, previously considered a mere byproduct, as a promising non-invasive diagnostic tool for early cancer detection due to their role in intercellular communication and transport of tumor-specific molecules. However, issues such as the lack of standardized protocols for isolation, characterization, analysis, and large-scale clinical trials must be addressed to ensure reliability and reproducibility. Phototherapy is another important aspect holding promise for cancer treatment; yet, the precise delivery of localized heat to tumor cells remains a significant challenge. In this regard, carbon carriers can be combined with photoactive molecules to enhance therapeutic outcomes and enable earlier detection (Tang et al., 2022). Further exploration of all mentioned techniques will promote enhanced nanotechnology in combating drug resistance faced by cancer therapeutics. The collaboration among scholars, healthcare professionals, engineers, and policymakers is essential for integrating nanotechnology into the global healthcare system. Lastly, investments in education, infrastructure, and technology transfer will expand nanotechnology’s potential in combating anticancer drug resistance and addressing future health challenges.

## 8 Conclusion

In conclusion, the complex challenge of drug resistance in cancer therapy presents a formidable hurdle that significantly diminishes the efficacy of conventional treatments. This resistance primarily arises from various mechanisms, including drug efflux, genetic mutations, epigenetic modifications, and the intricate tumor microenvironment (TME). The advent of nanotechnology undoubtedly offers a powerful promise in addressing these obstacles. The barriers posed by drug resistance can be mitigated through targeted drug delivery specificity and reduced systemic toxicity. The diverse range of nanoparticles, including liposomes, micelles, dendrimers, carbon-based materials, and metallic nanomaterials (NMs), has shown considerable potential in overcoming drug resistance across different cancer types. Furthermore, the integration of advanced technologies such as artificial intelligence in the design of NMs is revolutionizing therapeutic strategies. Although only a few nanotherapeutics have received clinical approval, it is crucial to address the ongoing challenges and limitations for successful clinical translation. Continuous investigation is essential to elucidate the molecular mechanisms underlying drug resistance to maximize the formulation and application of NMs in anticancer therapies. To enhance the potential of nanotechnology in combating cancer and improving patient outcomes, future initiatives should strengthen collaborations among researchers, clinicians, and policymakers.
